# Thermal inspection for viscous dissipation slip flow of hybrid nanofluid (TiO_2_–Al_2_O_3_/C_2_H_6_O_2_) using cylinder, platelet and blade shape features

**DOI:** 10.1038/s41598-023-34640-8

**Published:** 2023-05-23

**Authors:** Hong Yang, Umer Hayat, Shakil Shaiq, Azeem Shahzad, Tasawar Abbas, Muhammad Naeem, Sami Ullah Khan, Taher Labidi, Lioua Kolsi, Manzoor Ahmad Zahid

**Affiliations:** 1 School of Computer Science, Chengdu University, Chengdu, China; 2 Key Laboratory of Pattern Recognition and Intelligent Information Processing of Sichuan, Chengdu University, Chengdu, China; 3 Department of Basic Sciences, University of Engineering and Technology, Taxila, 47050 Pakistan; 4 Department of Mathematics, The Sahara University, Narowal, 51600 Pakistan; 5 Department of Mathematics, University of Wah, Wah Cantt, 47040 Pakistan; 6 Department of Mathematics, COMSATS University Islamabad Sahiwal Campus 57000, Pakistan, Sahiwal, Pakistan; 7 Department of Mathematics, Namal University, Mianwali, 42250 Pakistan; 8 Department of Software Engineering, College of Computer Engineering and Sciences, Prince Sattam Bin Abdulaziz University, P.O. Box 151, Al-Kharj, 11942 Saudi Arabia; 9 Miracl Laboratory, Sfax University, Sfax, Tunisia; 10 Department of Mechanical Engineering, College of Engineering, University of Ha’il, Ha’il City, 2440 Saudi Arabia; 11 Laboratory of Metrology and Energy Systems, Department of Energy Engineering, University of Monastir, 5000 Monastir, Tunisia

**Keywords:** Mathematics and computing, Nanoscience and technology

## Abstract

Hybrid nanofluid are the modified class of nanofluids with extra high thermal performances and present different applications in automotive cooling, heat transfer devices, solar collectors, engine applications, fusion processes, machine cutting, chemical processes etc. This thermal research explores the heat transfer assessment due to hybrid nanofluid with of different shape features. The thermal inspections regarding the hybrid nanofluid model are justified with aluminium oxide and titanium nanoparticles. The base liquid properties are disclosed with ethylene glycol material. The novel impact of current model is the presentation of different shape features namely Platelets, blade and cylinder. Different thermal properties of utilized nanoparticles at various flow constraints are reported. The problem of hybrid nanofluid model is modified in view of slip mechanism, magnetic force and viscous dissipation. The heat transfer observations for decomposition of TiO_2_–Al_2_O_3_/C_2_H_6_O_2_ is assessed by using the convective boundary conditions. The shooting methodology is involved for finding the numerical observations of problem. Graphical impact of thermal parameters is observed for TiO_2_–Al_2_O_3_/C_2_H_6_O_2_ hybrid decomposition. The pronounced observations reveal that thermal rate enhanced for blade shaped titanium oxide-ethylene glycol decomposition. The wall shear force reduces for blade shaped titanium oxide nanoparticles.

## Introduction

Recent advances in thermal engineering and nanotechnology claimed wide applications of nanomaterials in heat transfer devices, heavy industries, chemical processes, textile industries, biomedical applications, solar processes etc. The nanofluids are suspension of nanoparticles with base fluids like ethylene glycol, water, or silicone oil. In many engineering and industrial regime, such base materials are used as a source of energy. However, owing to low thermal impact and poor conducting performances, less heating outcomes are observed. Recent developments in nanotechnology, the thermal impact of several base fluids have been improved by using the interaction of nanoparticles. Due to intrinsic thermal characteristics like larger relative surface area and thermal conductivity, researchers have preferred nanoparticles to be best source of energy. The heat transfer and deferment strength are considerably increased when nanoparticles are suspended. Enhancing chiller heat transfer efficiency, domestic refrigerator freezers, cooling of electronics and transformer oil, boosting diesel generator efficiency, military and space, solar water heating, nuclear reactors, cooling of heat exchanging devices, and so on are only a few of the uses. Nanofluids have a wide range of applications because they can improve heat transfer performance when compared to pure liquids, earning them the moniker next-generation heat transfer fluids. Suspending metal particles in fluids to increase thermal conductivity is a well-known technique. Choi^1^ predicted first invention on nanomaterials and introduction the concept of nanofluids. Buongiorno^2^ presented the fascinating description related to slip mechanisms of the nanofluid that depends upon Brownian motion and thermophoresis characteristics. Hayat et al.^3^ presented suggestion about consideration of Maxwell nanofluid persuaded through the way of stretching surface which possessed variable thickness. Sui et al.^4^ proposed the investigations of modification of diffusion theories that can be used to analyze the phenomenon of mass and heat worked in Maxwell nanomaterial. Hsiao^5^ worked to theoretically investigate the flow of nanoparticles having characteristics of viscous dissipation and also thermal radiation. Turkyilmazoglu^6^ investigated the consequences of nanofluid flow having single phase in a circular jet. Ahmad et al.^7^ investigated the generation of heat and absorption characteristics in nanofluid of rate type persuaded by the rotating disk and the formulated problems are numerically solved by shooting method. For channel with a zero-mass flux rate, Turkyilmazoglu^8^ addressed the nanofluid thermal attention. Tlili et al.^9^ developed heating transfer model for assessing thermal determination of nanomaterials. Muhammad et al.^10^ focused on Carreau nanofluid heating impact via wedge configuration flow. Hayat et al.^11^ reported a nanofluid investigation associated to nanofluid flow. The chemical reactions of homogenous heterogeneous components seen in carbon nanotubes, which are made up of a rotating 3-D surface, were also studied by Hayat et al.^12^. Mahanthesh et al.^13^ investigated the nano materials which are magnetized SWCNT and MWCNT when there is a heat source with exponential growth. The importance of carbon nanotubes at the porous space is investigated by Muhammad et al.^14^. Magagula et al.^15^ have explored the bioconvection onset regarding the nanofluid continuation. Using enhanced Fick's and Fourier laws, Ibrahim et al.^16^ studied the mass and heat transfer processes for micropolar nanofluid with thermal impact. Acharya et al.^17,18^ have explored the mixed convection flow of carbon nanotubes organized by a moving curved surface. Rana and Gupta^19^ addressed the Stefan blowing aspect for rotating disk flow with nanofluid flow. Rana et al.^20^ tested the Hall features while addressing the enhanced heat transfer impact due to nanomaterials.

The hybrid nanomaterial is the more impressive class of nanofluid justifying the enhanced thermal mechanism. This hybrid class contains properties of base fluid with decomposition of two different nanoparticles. It is experimentally observed that more strengthened thermal performances are observed when heat transfer inspection is observed with hybrid nanofluids. Owing to such unique thermal impact, the special applications of hybrid class are noted in photovoltaic systems, thermal management systems, automotive cooling systems, energy storage devices, coating of materials, heat transfer objects etc. Different studies in recent years are predicted for hybrid nanofluids. Bibi et al.^21^ discussed the shape features of hybrid nanoparticles due to thin liquid layer. Saeed et al.^22^ endorsed the hybrid nanofluid thermal effectives due to spinning of moving regime. Ibrahim and Gamachu^23^ observed the entropy generation enrollment while inspecting the novel thermal outcomes of hybrid nanofluid. Alhadri et al.^24^ performed the computations by using the Response surface technique for a hybrid nanofluid problem conveying the classical heating impact. Dero et al.^25^ justified the stable thermal determination of hybrid nanofluid impacted by dissipation consequences. The shape features endorsing thermal classification of hybrid nanofluid was observed by Shanmugapriya et al.^26^. Mostafizur et al.^27^ depicted the solar application for hybrid nanofluid in cooling systems. Haneef et al.^28^ reported the progressive on set of hybrid nanoparticles via numerical approach. The gyrating channel surface flow containing the copper nanoparticles with ethylene glycol was invested by Das et al.^29^. Rana et al.^30^ reported the hybrid nanofluid flow due to copper nanoparticles with radiative heat flux. The role of quadratic thermal radiation for hybrid nanofluid with ethylene glycol base material for rotating sphere was focused in the model of Rana et al.^31^. Later on, Rana et al.^32^ worked out the Artificial neutral network framework for the hybrid nanofluid problem with elliptical fins. Ullah et al.^33^ explained the multiple shape consequences regarding the hybrid nanofluid with Darcy Forchheimer impact. Guedri et al.^34^ suggested the biomedical significance of aluminium and iron oxide nanoparticles against the blood base fluid. Mahmood et al.^35^ reported the Joule heating mechanism of hybrid nanoparticles with spinning sphere. Qadeer et al.^36^ continued the irreversibility mechanism of hybrid nanofluid in the divergent channel. Alqahtani et al.^37^ enrolled the dissipative fact for hybrid nanofluid with stretched disk.

This thermal research explores the heat transfer assessment due to hybrid nanofluid with of different shape features. Titanium oxide (TiO_2_) and aluminium oxide (Al_2_O_3_) tiny materials are used for endorsing the hybrid nanofluid thermal prospective. The justification of base liquid is observed with ethylene glycol (C_2_H_6_O_2_) liquid. The novel impact of current model is the presentation of different shape features namely Platelets, blade and cylinder. The microscopic view thermal visualization of nanoparticles is observed. Different thermal properties of hybrid nanofluid are observed with interreference of viscous dissipation, external magnetic force and heating source. The contribution of slip has also been observed for controlling of thermal transport phenomenon. The convective boundary assumptions are entertained for uprising the thermal impact. The shooting numerical technique is used to present the numerical outcomes. Various tables are developed for report the shape features assessment and thermal observations of proposed model. The enhanced thermal applications of ethylene glycol are important in different industrial and manufacturing processes.

## Hybrid nanofluid model

### Flow constraints and governing equations

**Figure 1:**
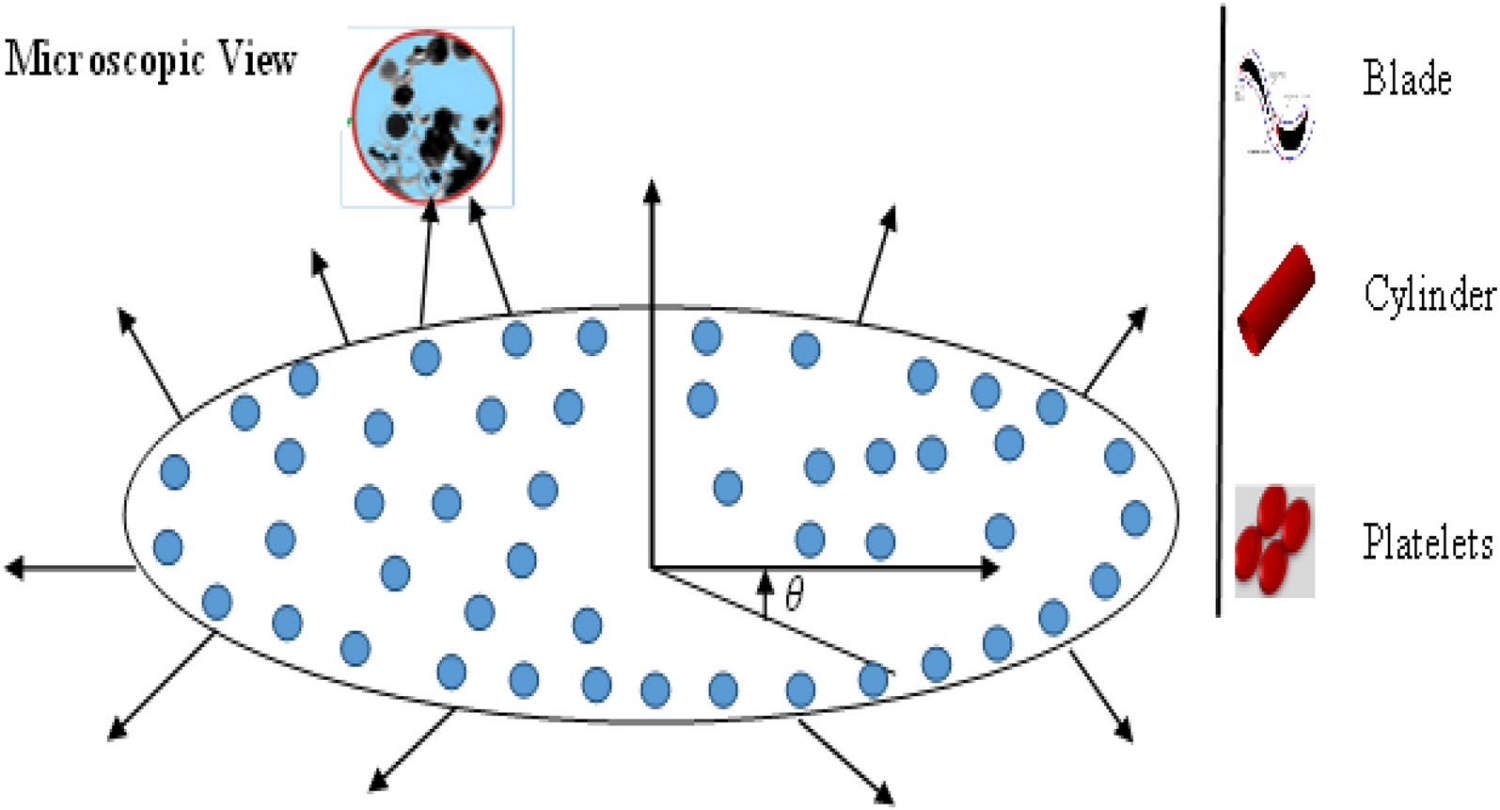
Schematic representation of the modelled problem.

The heat transfer enhancement due to hybrid nanofluid with different shape features is observed due to radially stretched surface. The nanofluid flow having velocity $U(r,t)=\frac{ar}{1-ct}$ occurs along a radial direction and pressure gradient plays no part in the fluid flow field. The surface temperature is assumed to be of form ${T}_{s}-{T}_{0}=-{T}_{r}\left[\frac{b{r}^{2}}{2{\nu }_{f}}\right]{\left(1-\alpha t\right)}^{-\frac{3}{2}},$ where ${T}_{0a}$ ambient temperature. The flow configuration is presented in Fig. 1. Following flow assumptions are assumed for model the problem:

- An unsteady 2-D hybrid nanofluid model in radially stretched surface framework is utilized.
- The flow is subject to the variable magnetic field. The role of induced magnetic force is neglected under the hypothesis of small magnetic Reynolds number hypothesis.
- Different shaped nanoparticles of $Ti{o}_{2} and A{l}_{2}{o}_{3}$ are submerged into ethylene glycol $(EG)$ base fluid for a comprehensive relative analysis.
- The effects of viscous dissipation and internal heat generation are considered to modify the problem.
- The mathematical structure is based on polar coordinate $(r, \theta , z)$.
- All physical entities are independent of $\theta$ against the rotational flow and the velocity field is in the form $v= [u(r,z), 0 , w(r,z)]$, where $u$ and $w$ are velocity components along the $radial (r)$ and $axial (z)$ directions, respectively.

The governing equations of problem are^21–23^:

1 $$\frac{\partial \dot{u}}{\partial \dot{r}}+\frac{\dot{u}}{\dot{r}}a+\frac{\partial \dot{w}}{\partial \dot{z}}a=0a,$$

2 $$\frac{\partial \dot{u}}{\partial \dot{t}}+\dot{u}\frac{\partial \dot{u}}{\partial \dot{r}}+\dot{u}\frac{\partial \dot{u}}{\partial \dot{z}}= \frac{{\mu }_{nf}}{{\rho }_{nf}}\frac{{\partial }^{2}\dot{u}}{\partial {\dot{z}}^{2}}-\frac{{\sigma }_{nf}}{{\rho }_{nf}}.{B}^{2}\left(t\right) \dot{u},$$

3 $$\frac{\partial \dot{T}}{\partial \dot{t}}+\dot{u}\frac{\partial \dot{T}}{\partial \dot{r}}+\dot{w}\frac{\partial \dot{T}}{\partial \dot{z}}={\alpha }_{nf}.\frac{{\partial }^{2}\dot{T}}{\partial {\dot{z}}^{2}}+\frac{{\mu }_{nf}}{{\left(\rho {C}_{p}\right)}_{nf}}a{\left(\frac{\partial \dot{u}}{\partial \dot{z}}\right)}^{2}+\frac{{Q}_{0}}{{\left(\rho {C}_{p}\right)}_{nf}}\left(\dot{T}-{\dot{T}}_{\infty }\right)$$

The corresponding boundary conditions for the given system of differential equations are^23^:

4 $$\left\{\begin{array}{c}\dot{u}=U+K\frac{\partial \dot{u}}{\partial \dot{z}},\dot{w}=0,-{k}_{nf}\left(\frac{\partial \dot{T}}{\partial \dot{z}}\right)={k}_{f}\left({\dot{T}}_{f}-{\dot{T}}_{\infty }\right), \,at\, \dot{z}=0, \\ \dot{u}\to 0,\dot{T}\to {\dot{T}}_{\infty }, \,as\, \dot{z}\to \infty .\end{array}\right.$$

where

$$a{\alpha }_{nf}=\frac{{K}_{nf}}{{\left(\rho {C}_{p}\right)}_{nf}}a , \,\,a{\rho }_{nf}=\left(1-\phi \right)a{\rho }_{f}+\phi a{\rho }_{s},$$

$${\mu }_{nf}= {\mu }_{f}\left(1+{D}_{1}\phi +{D}_{2}{\phi }^{2}\right)a,{\sigma }_{nf}={\sigma }_{f}.\left(1-\phi \right)+\phi {.\sigma }_{s},$$

5 $$a\frac{{K}_{nf}}{{k}_{f}}a =a\frac{{k}_{s }+\left(mm - 1\right){k}_{f} + \left( mm-1 \right).\left({ k}_{s}-{ k}_{f}\right).\phi }{{k}_{s} +\left( m m-1 \right).{ k}_{f}-\left({ k}_{s}-{ k}_{f}\right).\phi },$$

With density ${(\rho }_{nf})$, thermal diffusivity $({\alpha }_{nf})$, volume fraction $\phi$, thermal conductivity $({k}_{s})$ , shape factor of nanoparticles $(nm)$, viscosity $({v}_{nf} )$ and electrical conductivity $({\sigma }_{nf})$, thermal effeciency ${k}_{nf}$ and viscosity (${\mu }_{nf}$). Moreover,$\phi , {(\rho Cp)}_{nf}, {D}_{1},\text{ and } D_2$ are volume fraction, heat capacitance and viscosity enhancement coefficients of the nanofluid, respectively.

### Thermal features and shape features of hybrid nanoparticles

The characteristics of $Ti{O}_{2} \,and \,A{l}_{2}O$ tiny materials with $EG$ base material are reflected via Table 1. The shape factor and viscosity of different shapes nanoparticles are given in Table 2.

**Table 1:** Characteristics of ${\mathrm{Tio}}_{2}\mathrm{ and A}{\mathrm{l}}_{2}{\mathrm{o}}_{3}$ nanoparticles and base fluid^21^.

Base fluid/nanoparticles	$\mathrm{Specific heat}$ ${\dot{\mathrm{C}}}_{\dot{\mathrm{p}}}(\mathrm{J}$/$\mathrm{g Ka})$	$\mathrm{ThermalConductivity}$ $\dot{\mathrm{K}}$ $(\mathrm{W}$/$\mathrm{mK})$	Density $\dot{\uprho }$ $(\mathrm{kg}$/${\mathrm{m}}^{3})$	Electrical conductivity $\dot{\upsigma } (\mathrm{S}/\mathrm{m})$
*Ethylene Glycol (EG)*	2430	*0.253*	*1115*	*3.14*
Titanium $(Ti{o}_{2})$	686.2	8.9538	4250	0.125
*Aluminum* $(A{l}_{2}{o}_{3})$	765	*40*	*3970*	*16.5*

**Table 2:** Numerical visualization of shape factor and viscosity of nanoparticles^21^.

Parameters/nanoparticles			
${D}_{1}$	$14.6$	$13.5$	$37.1$
${D}_{2}$	$123.3$	$904.4$	$612.6$
$mm$	$8.26$	$4.82$	$5.72$

### Dimensionless variables

Endorsing the new variables^21–23^:

6 $$\left.\begin{array}{c}\psi ={\dot{r}}^{2}U R{e}^{- \frac{1}{2}}. f\left(\eta \right),\\ \theta \left(\eta \right)=\frac{ {(\dot{T}}_{0 }- \dot{T})}{{T}_{ref}. \left(\frac{b{\dot{r}}^{2}}{2v{\left(1-\alpha \dot{t}\right)}^\frac{3}{2}}\right)}\\ \eta =\frac{\dot{z}}{\dot{r}} R{e}^\frac{1}{2}\end{array}\right\}$$

where $\upeta$ is an independent variable, $aRe=.\frac{\dot{r}U}{{v}_{f}}$ is a local Reynolds number, and $\uppsi$ is a stream function

7 $$a\dot{u}=-\frac{1}{\dot{r}}a\frac{\partial \psi }{\partial \dot{z}} ,a\dot{w}a=\frac{1}{\dot{r}}.\frac{\partial \psi }{\partial \dot{r}}$$

As a result, the velocity components are calculated as:

8 $$a\dot{u}a=aU{f}{^{\prime}}\left(\eta \right)a,\; \mathrm{ and }\quad a\dot{w}a=-a2UaR{e}^{- \frac{1}{2}} f\left(\eta \right)$$

By utilizing the relationship defined in Eqs. (6) to (8), Equation (1) is identically satisfied and equations (2) and (3) along with boundary conditions defined in Eq. (4) take the following form:

9 $${f}^{{{\prime}}{{\prime}}{{\prime}}}-a\frac{{\epsilon }_{4}}{{\epsilon }_{1}}M.{f}{^{\prime}}-\frac{1}{{\epsilon }_{1}}aS.\left({f}{^{\prime}}+ \frac{\eta }{2}a{f}^{{{\prime}}{{\prime}}}\right)-\frac{1}{{\epsilon }_{1}}{a\left({f}{^{\prime}}\right)}^{2}+\frac{2}{{\epsilon }_{1}}af.{f}^{{{\prime}}{{\prime}}}a=0,$$

10 $${\theta }^{{{\prime}}{{\prime}}}+\frac{Pr}{{\epsilon }_{2}}.Ec.{\epsilon }_{1}{{(f}^{{{\prime}}{{\prime}}})}^{2}-\frac{Pr}{{\epsilon }_{2}}.\left(\frac{S}{2}\left(3\theta +\eta {\theta }{^{\prime}}\right)-2 {f}{^{\prime}}\theta +2f{\theta }{^{\prime}}\right)-\frac{1}{{\epsilon }_{2}}Pr.Q \theta =0.$$

Subject to the boundary conditions

11 $$\left.\begin{array}{c}f.\left(0\right)=.0, {f}{^{\prime}}\left(0\right)=.1+K {f}^{{{\prime}}{{\prime}}}\left(0\right), \\ { \theta }{^{\prime}}\left(0\right)=- \frac{{k}_{f}}{{k}_{nf}}.Bi.\left(1-\theta \left(0\right)\right) , {f}{^{\prime}}\left(\infty \right), \theta \left(\infty \right).=0. \end{array}\right\}$$

where, ${\upepsilon }_{1},{\upepsilon }_{2},{\upepsilon }_{3} \,and\, {\upepsilon }_{4}$ are constants which are defined as^25^

12 $$\left\{\begin{array}{l}{\epsilon }_{1}=\frac{1 + {D}_{1}\cdot \phi +{ D}_{2}\cdot {\phi }^{2}}{1-\phi + \phi \cdot \left(\frac{{\rho }_{s}}{{\rho }_{f}}\right)}, {\epsilon }_{2}=\left(1-\phi +\phi \left(\frac{{\left(\rho \cdot {C}_{p}\right)}_{s}}{{\left(\rho {\cdot C}_{p}\right)}_{f}}\right)\right),\\ {\epsilon }_{3}=\frac{1}{{\epsilon }_{2}}\cdot \frac{{k}_{f}}{{k}_{nf}}, {\upepsilon }_{4}=\cdot \frac{1-\upphi +\upphi \cdot \left(\frac{{\upsigma }_{\mathrm{s}}}{{\upsigma }_{\mathrm{f}}}\right)}{1-\upphi +\upphi \cdot \left(\frac{{\uprho }_{\mathrm{s}}}{{\uprho }_{\mathrm{f}}}\right)}\cdot \end{array}\right.$$

The non-dimensional physical parameters such like magnetic number $M$, internal heat generation $Q$, Unsteadiness parameter $S$, Prandtl number $Pr$, Eckert number $Ec$ and Biot factor $Bi$ are defined as:

13 $$M=\frac{{\sigma }_{f}.{B}_{0.}^{2}\dot{r}}{{\rho }_{f.}U}, Pr=\frac{{\nu }_{f.}{\left(\rho .{C}_{p}\right)}_{f}}{{k}_{f}}, Ec=\frac{{U}^{2}}{{C}_{p}.({T}_{w}.-.{T}_{0})}, Q=\frac{{Q}_{0.}r}{U{\left(\rho .{C}_{p}\right)}_{f}}, S=\frac{.c}{a},Bi= \frac{{h}_{f}}{{k}_{f}}.\sqrt{\frac{{\nu }_{f}r}{{U}_{w}}}$$

Shear stress and Heat transfer rate can be defined as:

14 $${C}_{f\dot{r}}=\frac{{\tau }_{w}}{\rho .{\left({\dot{u}}_{w}\right)}^{2}}, N{u}_{\dot{r}}=\frac{\dot{r}{q}_{\dot{w}}(\dot{r})}{{k}_{f}.\left[{T}_{f}-{T}_{\infty }\right]},{\tau }_{w}={\mu }_{nf}.{\left.\frac{\partial \dot{u}}{\partial \dot{z}}\right|}_{\dot{z}=0},{q}_{\dot{w}}\left(\dot{r}\right)=-{k}_{nf}.{\left(\frac{\partial \dot{T}}{\partial \dot{z}}\right)}_{\dot{z}=0}$$

The dimensionless form is:

15 $${C}_{f}{ Re}^\frac{1}{2}=\left(1+{D}_{1}.\varphi +{D}_{2}.{\varphi }^{2}\right).{f}^{{{\prime}}{{\prime}}}\left(0\right), Nu {Re}^{-\frac{1}{2}}= -\frac{{k}_{nf}}{{k}_{f}}.{\theta }^{^{\prime}}\left(0\right).$$

## Numerical simulations

The numerical outcomes are preserved via BVP4C algorithm. The first order system is:

$$\left\{\begin{array}{l}f=.{y}_{\left(.1\right)}, \\ {y}_{\left(.1\right)}^{^{\prime}}.=.{y}_{\left(.2\right)}, \\ {y}_{\left(.2\right)}^{^{\prime}}.=.{y}_{\left(.3\right)} \\ {y}_{(.3)}^{^{\prime}}=\frac{1}{{\epsilon }_{1}}\left({\epsilon }_{4 }.{M.y}_{\left(.2\right)}\right)+\frac{1}{{\epsilon }_{1}}\left(S.\left({y}_{\left(.2\right)}+\frac{\eta }{2}.{y}_{\left(3\right)}\right)+{\left({y}_{\left(.2\right)}\right)}^{2}-2 {y}_{\left(1\right)}{y}_{\left(2\right)}\right),\end{array}\right.$$

16 $$\left\{\begin{array}{l}\theta ={y}_{\left(4\right)}, \\ {{y}^{^{\prime}}}_{\left(4\right)}={y}_{\left(5\right)}, \\ {y}_{(5)}^{^{\prime}}=\frac{Pr}{{\epsilon }_{2}}\left(\frac{S}{2}\left(3{y}_{(4)}+\eta {y}_{(5)}\right)+2 {y}_{(2)}{y}_{(4)}-2{y}_{(1)}{y}_{(5)}-Ec{\epsilon }_{1}{\left({y}_{\left(3\right)}\right)}^{2}\right)-\frac{Pr}{{\epsilon }_{2}}Q {y}_{(4)},\end{array}\right.$$

with:

17 $$\left.\begin{array}{c}{\mathrm{y}}_{(.1)}\left(0\right)=0, {\mathrm{ y}}_{(.2)}\left(0\right)=1+K {\mathrm{y}}_{\left(.3\right)}\left(0\right),{\mathrm{y}}_{\left(.5\right)}\left(.0\right)=-\frac{{k}_{f}}{{k}_{nf}}.Bi.\left(1-{y}_{\left(4\right)}\left(0\right)\right) \\ {\mathrm{y}}_{(.3)}\left(\infty \right)=0, {\mathrm{y}}_{(.5)}\left(\infty \right)=0 \end{array}\right\}$$

The numerical results are presented with accuracy of 10^–4^.

## Thermal impact of parameters

A numerical solution of the dimensionless mathematical model is obtained using the BVP4C technique. Significant parameters such as the $M$ magnetic parameter, $S$ unsteadiness parameter, $\phi$ solid volume fraction, $Ec$ Eckert number, $Bi$ Biot number and $Q$ internal heat generation parameter is all given special attention. In addition, there is a detailed result for skin friction and heat transfer coefficient.

### Assessment of velocity profile

**Figure 2:**
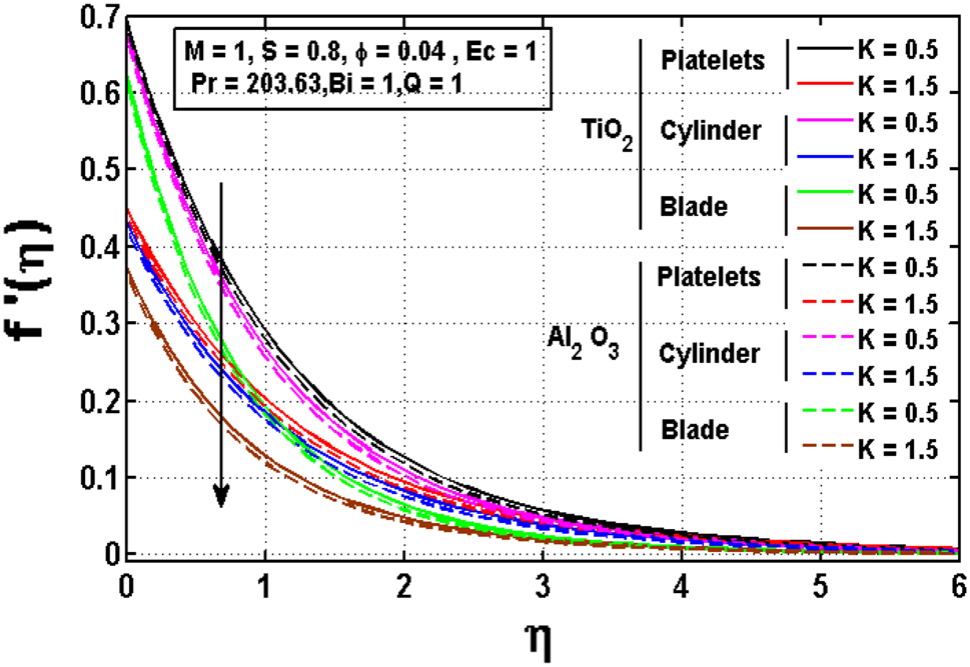
Impact of $K$ on velocity profile.

**Figure 3:**
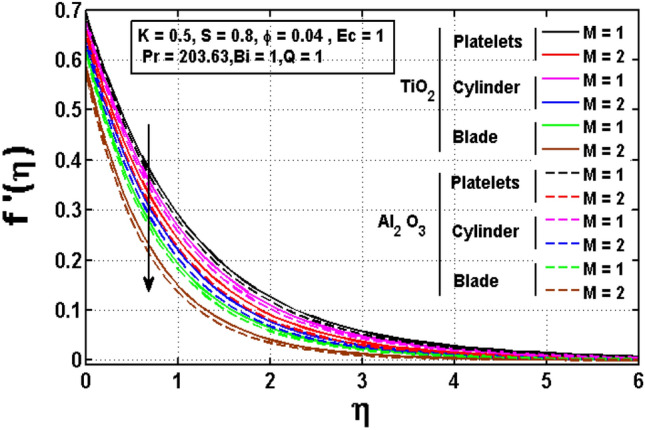
Impact of $M$ on velocity profile.

**Figure 4:**
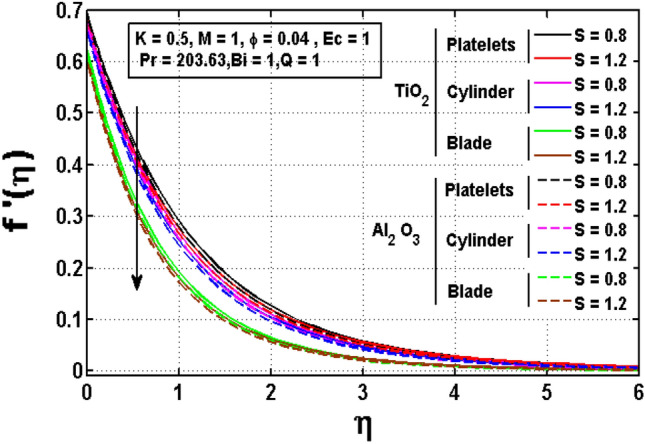
Impact of $S$ on velocity profile.

Figures 2, 3, 4 are drawn to investigate the influence of significant physical parameters such as velocity slip parameter $K,$ Magnetic parameter $M$ and unsteadiness parameter $S$ on velocity distribution. Figure 2 signifies the assessment of slip factor $K$ on velocity field for various shapes of $Ti{O}_{2} \mathrm{and }A{l}_{2}{O}_{3}$ anoparticles dispersed in ethylene glycol $(EG)$. Physically, the dragging of the stretching sheet is only partially coupled to the fluid under the slip parameter. The slip velocity increases while fluid velocity drops as $K$ increases. It is worth noting that $K$ has significant influence on the solutions. Furthermore, the enhancing and declining rate of velocities are noted for platelet shaped $Ti{O}_{2}-EG$ and Blade shaped $A{l}_{2}{O}_{3}-EG$ nanofluids. Figure 3 impacted the magnetic parameter association to velocity profile of two distinct nanoparticles $Ti{O}_{2} \mathrm{and }A{l}_{2}{O}_{3}$ for different shapes such as blade, cylinder and platelet. It is noticed that greater values of the magnetic parameter generate more Lorentz force, which causes the fluid velocity to slow and the velocity profile to drop. Figure 4 illustrates fluctuated pattern in velocity for *S*. The movement of the boundary layer thickness is slowed as $S$ increases, resulting in a decrease in velocity profile for both type of nanofluids having various shapes of $Ti{O}_{2}$ and $A{l}_{2}{O}_{3}$ nanoparticles.

### Assessment of temperature profile

**Figure 5:**
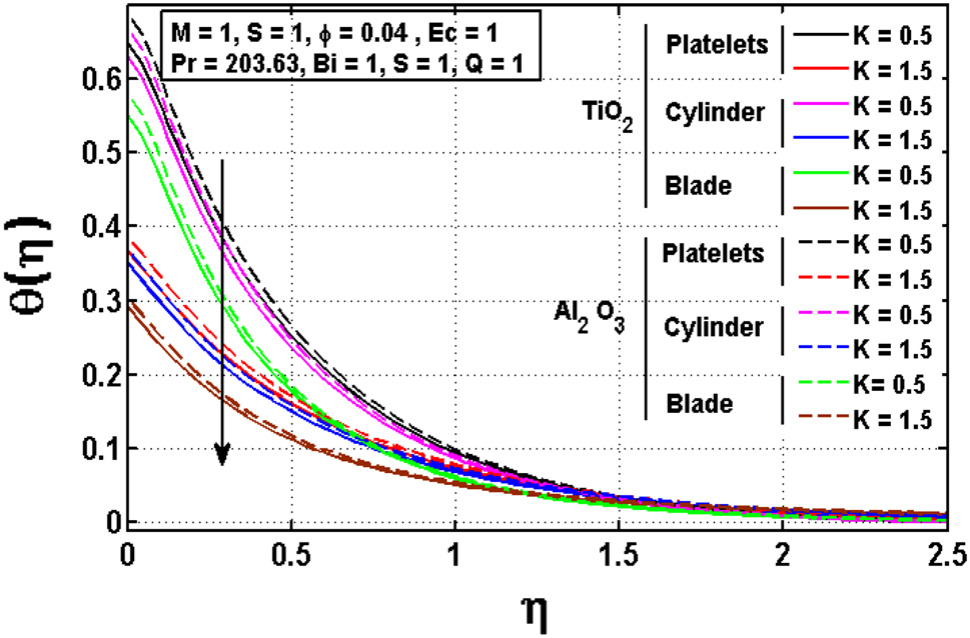
Impact of $K$ on temperature profile.

**Figure 6:**
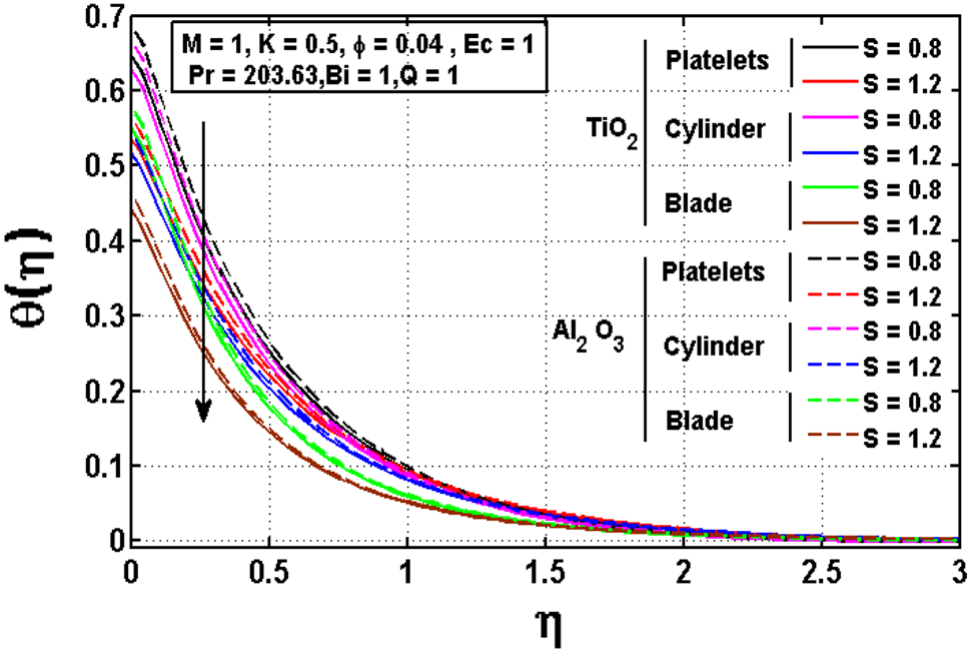
Impact of $S$ on temperature profile.

**Figure 7:**
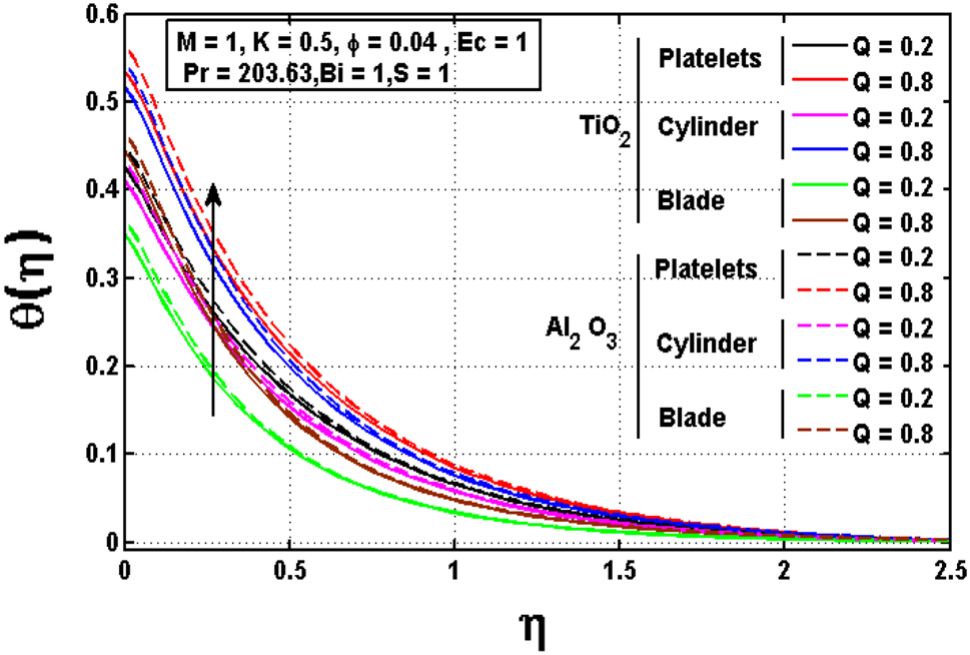
Impact of $Q$ on temperature profile.

**Figure 8:**
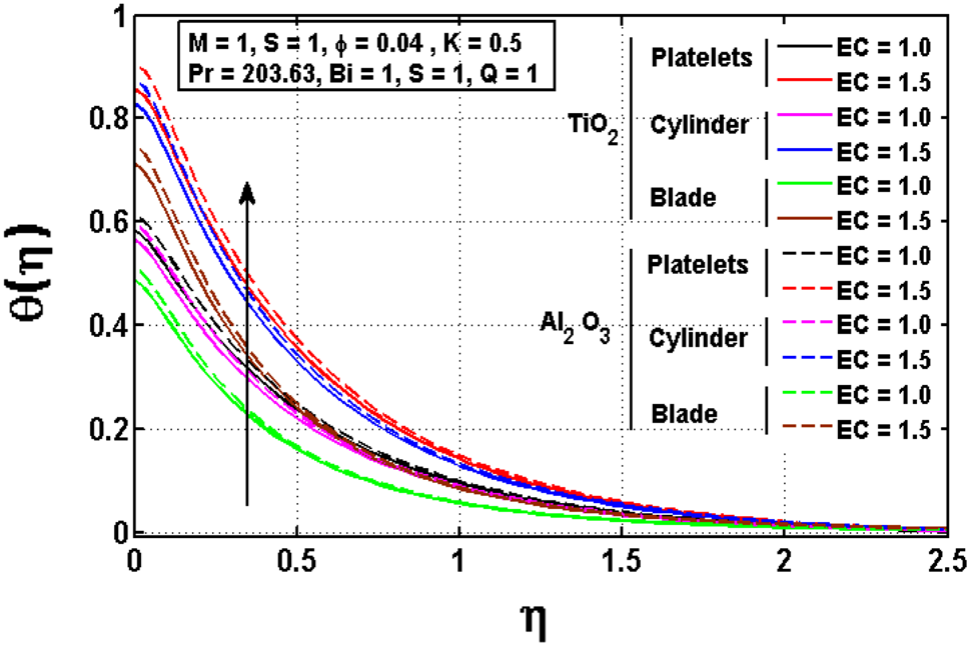
Impact of $Ec$ on temperature profile.

**Figure 9:**
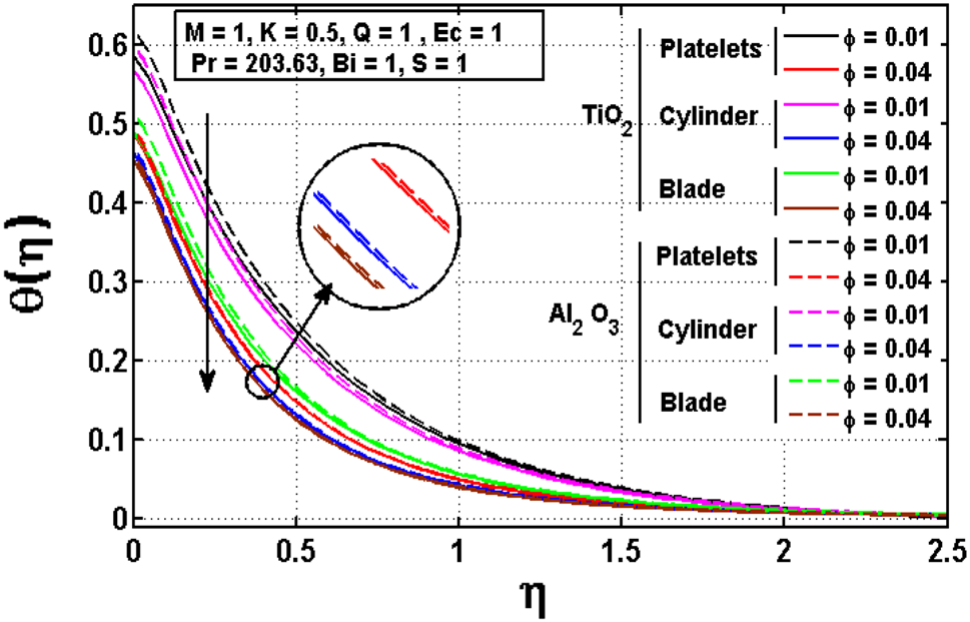
Impact of $\phi$ on temperature profile.

**Figure 10:**
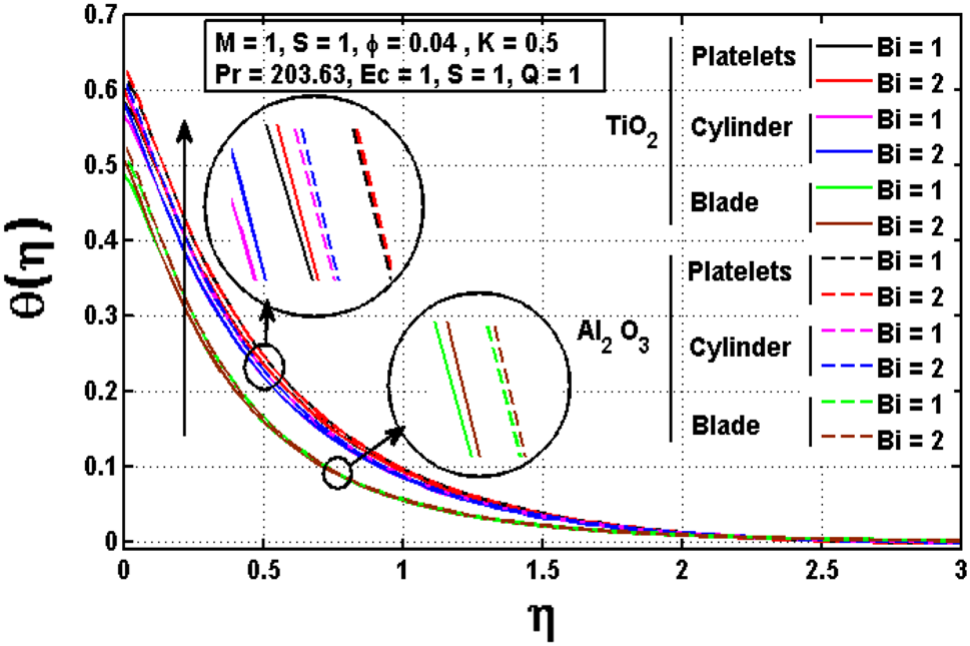
Impact of $Bi$ on temperature profile.

This sub section is reserved to discuss the variation of significant physical parameters such as unsteadiness parameter $\mathrm{S}$, internal heat generation parameter $\mathrm{Q}$, Eckert number $\mathrm{EC},$ slip parameter $K$ and Biot constant $\mathrm{Bi}$ on temperature $\theta$. The variation appeared in $\theta$ for $K$ are regarded in Fig. 5. The decrement in $\theta$ for arises impact of $K$ is noted. Maximum temperature is witnessed for platelets shaped $A{l}_{2}{O}_{3}-EG$ nanofluid and minimum temperature is observed blade shaped $Ti{O}_{2}-EG$ nanofluid. Figure 6 exhibits the impact of $S$ for $\theta$. The heat transfer decreases when the unsteadiness parameter increases. It means unsteadiness significantly influence the temperature profile. Figure 7 depicts the effect of the internal heat generating parameter $Q$ on temperature profile. It is observed that boosted onset of $\theta$ is noticed for larger $Q.$ It is due to the fact that positive values of $Q$ shows the temperature storage within the fluid that plays a vital role to enhance the fluid temperature. The viscous dissipation impact on temperature is visualized in Fig. 8. The temperature increases for rising values of $EC$ for $A{l}_{2}{O}_{3}-EG$ and $Ti{O}_{2}-EG$ nanofluids. The increase in viscous dissipation indicates enhancement in the kinetic energy in the fluid which improves the temperature distribution. Figure 9 depicts the influence of volumetric fraction $\phi$ on $\theta$. The rescaling values to $\phi$ declined the thermal phenomenon for $A{l}_{2}{O}_{3}-EG$ and $Ti{O}_{2}-EG$. Such observations are associated to the rising values of $\phi$ thermal conductivity which rapidly transfer the. Figure 10 illustrates the influence of Biot number $Bi$ on temperature profile. It is observed that temperature gradient for both $BiA{l}_{2}{O}_{3}-EG$ and $Ti{O}_{2}-EG$ nanofluids upsurges for $Bi$. Such enhanced inspection is due to larger heat transfer coefficient.

### Variation of skin friction

Table 3 shows the influence of parameters like $S$, $M$ and $\mathrm{K}$ on skin friction for multi-shape $Ti{O}_{2}and A{l}_{2}\mathrm{O}$ nanoparticles. The analysis of this table reveals that grad force enhanced when $S$ and $M$ increases, whereas the opposite trend is witnessed as the slip parameter $(\mathrm{K})$ increases. Moreover, the minimum value of skin friction is noted for blade shaped $Ti{O}_{2}$ nanoparticles whereas the maximum value of skin friction is observed for platelets shaped $A{l}_{2}{O}_{3}$. The magnitude of wall shear force is comparatively larger for platelets shaped nanoparticles.

**Table 3:** Absolute values of Skin friction coefficients ${{\varvec{C}}}_{{\varvec{f}}}{\varvec{R}}{{\varvec{e}}}^{\frac{1}{2}{\varvec{a}}}$.

Physical parameters			Skin friction ${C}_{f}R{e}^\frac{1}{2}$					
			Blade		Cylinder		Platelets	
$\mathbf{S}$	${\varvec{K}}$	**M**	$A{l}_{2}{O}_{3}$	$Ti{O}_{2}$	$A{l}_{2}{o}_{3}$	$Ti{O}_{2}$	$A{l}_{2}{O}_{3}$	$Ti{O}_{2}$
0.8	0.5	1	1.3630139	1.3341228	1.9546213	1.9113178	2.1625582	2.1142758
1.2	–		1.4087687	1.3828061	2.0238434	1.9844815	2.2399708	2.1959287
0.8	0.5		1.3630139	1.3341228	1.9546213	1.9113178	2.1625582	2.1142758
–	1.5		0.75258844	0.74142636	1.1482435	1.129644	1.2931561	1.271809
–	0.5	1	1.3630139	1.3341228	1.9546213	1.9113178	2.1625582	2.1142758
–	–	2	1.508916	1.4632767	2.1784294	2.1078508	2.4139072	2.3344361

### Variation of Nusselt number

Table 4 is drawn to analyze the influence of significant parameters such as unsteadiness parameter $S,$ Volumetric fraction $\phi$, Biot Number $Bi$, Internal heat generation parameter $Q$ velocity slip parameter $K,$ Eckert number $EC$, and magnetic parameter $M$ on heat transfer rate (Nusselt Number) for several types of $Ti{O}_{2} and A{l}_{2}{O}_{3}$ nanoparticles at the stretching sheet. The Nusselt number gets larger impact for $S$ and Biot number $Bi$ whereas the opposite trend is seen for rising values of $\phi , Q, Ec, K$ and $M$. The maximum and minimum heat transfer rate is noted for blade shape $Ti{O}_{2}$ and platelets shape $A{l}_{2}{O}_{3}$ nanoparticles.

**Table 4:** Numerical impact of parameters on Nusselt number.

Physical parameters							Nusselt number					
							Blade		Cylinder		Platelets	
$S$	$\phi$	$Bi$	*Q*	$K$	$Ec$	*M*	$A{l}_{2}{O}_{3}$	$Ti{O}_{2}$	$A{l}_{2}{O}_{3}$	$TiO$	$A{l}_{2}{O}_{3}$	$Ti{O}_{2}$
0.8	0.04	1	1	0.5	1	1	0.41813742	0.44668908	0.3202711	0.35589442	0.29390603	0.33086777
1.2							0.53804524	0.55721432	0.4516043	0.47660841	0.42820889	0.45439778
1	0.01	1	1	0.5	1	1	0.54067748	0.54956808	0.53057269	0.53630735	0.50590621	0.51200929
	0.05						0.35067748	0.34597573	0.39472845	0.42425464	0.36996315	0.40076182
1	0.04	1	1	0.5	1	1	0.48646833	0.50956808	0.39472845	0.42425464	0.36996315	0.40076182
		2					0.9349136	0.97877348	0.75837198	0.81496684	0.71146756	0.77048799
1	0.04	1	0.2	0.5	1	1	0.633045	0.64870263	0.56211592	0.58272	0.54283371	0.56445676
			0.8				0.53384633	0.55446745	0.44817081	0.47479183	0.42499186	0.45281787
1	0.04	1	1	0.5	1	1	0.41813742	0.44668908	0.3202711	0.35589442	0.29390603	0.33086777
				1.5			0.69478715	0.70664878	0.62504515	0.64173978	0.60480986	0.62248853
1	0.04	1	1	0.5	1	1	0.48766547	0.51106452	0.40243184	0.43324958	0.38211366	0.41470641
					1.5		0.25182686	0.28720713	0.12411659	0.1704225	0.093122824	0.14214634
1	0.04	1	1	0.5	1	1	0.41813742	0.44668908	0.3202711	0.35589442	0.29390603	0.33086777
						2	0.27331182	0.32399468	0.14187363	0.20643105	0.10678928	0.17444526

## Conclusions

The heat transfer analysis due to modified hybrid nanofluid has been numerically studied due to radially stretching sheet. The aluminium oxide and titanium nanoparticles are used with ethylene glycol base material. The blade, cylinder and platelets shaped nanoparticles are focused. The analysis has been observed in view internal heat generation, viscous dissipation and velocity slip effects. The significant observations are:

- With larger unsteady parameter, the velocity pattern reduced.
- The increasing velocity rate is claimed for platelet shaped titanium oxide-ethylene glycol suspension.
- The nanoparticles volumetric friction, velocity slip parameter and unsteadiness parameter reduce the heat transfer phenomenon.
- The internal heat generation, viscous dissipation and convective boundary conditions are seen to increase the nanofluid temperature.
- The unsteadiness parameter and Biot number effectively improves the heat transfer rate.
- The magnitude wall shear force controls due to slip parameter.
- The titanium oxide nanoparticles with a blade shape present enhanced heat transfer rate.
- The wall shear force reduces due to titanium oxide nanoparticles with blade shape.
- The blade shape features for titanium oxide and ethylene glycol decomposition are more useful for producing unsteady radially modules.

## Data Availability

The datasets generated/produced during and/or analyzed during the current study/research are available from the corresponding author on reasonable request.
